# Observation-based assessment of secondary water effects on seasonal vegetation decay across Africa

**DOI:** 10.3389/fdata.2022.967477

**Published:** 2022-09-09

**Authors:** Çağlar Küçük, Sujan Koirala, Nuno Carvalhais, Diego G. Miralles, Markus Reichstein, Martin Jung

**Affiliations:** ^1^Department of Biogeochemical Integration, Max Planck Institute for Biogeochemistry, Jena, Germany; ^2^Hydro-Climate Extremes Lab (H-CEL), Faculty of Bioscience Engineering, Ghent University, Ghent, Belgium; ^3^Center for Environmental and Sustainability Research (CENSE), Departamento de Ciências e Engenharia do Ambiente, Faculdade de Ciências e Tecnologia, Universidade NOVA de Lisboa, Caparica, Portugal

**Keywords:** ecohydrology, Africa, water limitation, groundwater, topography, secondary water resources, vegetation decay rate, drylands

## Abstract

Local studies and modeling experiments suggest that shallow groundwater and lateral redistribution of soil moisture, together with soil properties, can be highly important secondary water sources for vegetation in water-limited ecosystems. However, there is a lack of observation-based studies of these terrain-associated secondary water effects on vegetation over large spatial domains. Here, we quantify the role of terrain properties on the spatial variations of dry season vegetation decay rate across Africa obtained from geostationary satellite acquisitions to assess the large-scale relevance of secondary water effects. We use machine learning based attribution to identify where and under which conditions terrain properties related to topography, water table depth, and soil hydraulic properties influence the rate of vegetation decay. Over the study domain, the machine learning model attributes about one-third of the spatial variations of vegetation decay rates to terrain properties, which is roughly equally split between direct terrain effects and interaction effects with climate and vegetation variables. The importance of secondary water effects increases with increasing topographic variability, shallower groundwater levels, and the propensity to capillary rise given by soil properties. In regions with favorable terrain properties, more than 60% of the variations in the decay rate of vegetation are attributed to terrain properties, highlighting the importance of secondary water effects on vegetation in Africa. Our findings provide an empirical assessment of the importance of local-scale secondary water effects on vegetation over Africa and help to improve hydrological and vegetation models for the challenge of bridging processes across spatial scales.

## 1. Introduction

Drylands cover more than 40% of the land surface globally (D'Odorico et al., 2019). They have a strong impact on the global carbon cycle (Lal, 2019) and are sensitive to large interannual climatic variations (Brandt et al., 2018). Furthermore, more than one-third of the World's population is settled on drylands (Reynolds et al., 2007), 90% of which are in developing countries that are highly dependent on ecosystem services (Maestre et al., 2012). Despite their importance, the ecohydrology of the drylands is still not well understood (Maestre et al., 2021). This is particularly the case in Africa, where drylands cover 75% of the surface, yet, they remain largely understudied (Maestre et al., 2012; Adole et al., 2016; Prăvălie, 2016).

Apart from precipitation as the primary supply of water on the land, secondary water effects such as groundwater (Fan, 2015; Maxwell and Condon, 2016), capillary rise (Koirala et al., 2019), and lateral flows at hillslope scales (Fan et al., 2019), could be essential for vegetation in drylands (Miguez-Macho and Fan, 2021). Despite calls to better incorporate such complex interactions (Fan et al., 2007; Kollet and Maxwell, 2008), land surface models still lack representations that can capture these secondary but non-trivial effects of the water cycle on vegetation (Van Dijk et al., 2018; Mu et al., 2021). The challenge is mainly on the representation of local-scale land surface heterogeneity and associated hydrological processes at a relatively coarser spatial resolution of the Earth System Models (Clark et al., 2015; Fisher and Koven, 2020; Bly et al., 2021). Unfortunately, to the best of our knowledge, there are also no observation-based studies on the relevance of such secondary water effects on vegetation over large spatial domains that would aid the formulation and development of model processes.

While it is not feasible to observe the relevant water fluxes and storages at a fine spatiotemporal resolution over large areas directly, remote sensing facilitates detailed monitoring of vegetation dynamics, which would be modulated by, among other factors, secondary water, providing imprints of the importance of secondary moisture for vegetation dynamics. For instance, the impact of secondary water on vegetation can be expected to be the largest in periods of progressive water limitation. Küçük et al. (2022) showed that vegetation cover decay is controlled by water availability to the first order over most of Africa, consistent with previous literature, and with theoretical expectations of dryland ecohydrology (Rodriguez-Iturbe and Porporato, 2005). The secondary moisture effects essentially act to keep water longer in the system and fuel plant accessible soil moisture for a prolonged period, which results in a delayed and buffered decay of the vegetation cover. It should, though, be noted that secondary water inputs are unlikely to be the dominant control of dry season vegetation cover decays across the continent, where the large scale patterns should be primarily related to climate regimes and vegetation characteristics. Thus, the main objective of this study is to isolate and attribute the effects of secondary water on vegetation cover decay that are not explained by the main climate gradients.

In recent years, Machine Learning (ML) has provided great opportunities for data-driven modeling of complex patterns and interactions in large Earth observation datasets despite the challenges with interpretability of these models (Rudin et al., 2022). Developments in interpretable ML are now shedding light on the “black box” models that characterize artificial intelligence algorithms (Molnar, 2019). This allows for attributing the contributions of input variables to target variables of an ML model and provides unprecedented opportunities in understanding land surface processes using state-of-the-art Earth Observation datasets.

In this study, we quantify the effect of terrain properties—as variables associated with local-scale moisture convergence and secondary water—in the seasonal decay rate of vegetation cover (λ) from remote-sensing observations over African drylands. Given the association of λ with a wide range of vegetation and climatic characteristics, the role of local-scale availability of secondary water on producing the spatial patterns of vegetation decay rate is yet to be demonstrated. Therefore, in order to quantify the effects of non-climatic water inputs on water-limitation induced ecosystem decay rate, we model λ using climate, vegetation, and terrain properties from an array of data products using interpretable ML. We first present a quantitative map of the importance of terrain properties, associated with the effects of secondary water, across Africa. We further investigate the conditions which enhance the relevance of secondary water on vegetation cover dynamics over Africa.

## 2. Data and methods

### 2.1. Seasonal decay rate of vegetation cover

In this study, we investigate the drivers of the spatial variations of the seasonal decay rate of vegetation cover, λ, which is estimated using an asymptotic exponential decay function across Africa using daily geostationary satellite retrievals of 16-year long Fractional Vegetation Cover data at ca. Five kilometer spatial resolution (Küçük et al., 2022). The asymptotic decay function quantifying the decay rate, independent of amplitude and timing of the event, allows comparing the rate of decay of vegetation across different climate zones, thus understanding the driving factors behind the spatial variation of λ. Initial analysis of λ showed that λ corroborates the rate of decrease in plant available water use under water limited conditions (Küçük et al., 2022). For a given level of aridity, a taller canopy decays more slowly, thus larger λ values, than a shorter one, which agrees with the previous field-based studies (Teuling et al., 2006; Boese et al., 2019). Moreover, variation of λ in relation to tree cover and aridity reflects plant adaptation strategies against water limitation, i.e., strong ecosystem-scale drought coping strategies in drought-stressed forests and savannahs (Singh et al., 2020). Therefore, apart from the first order climate-driven gradients at continental scales, λ contains information on secondary processes that affect vegetation decay in local-scales and presents opportunities to understand local-scale processes across Africa.

### 2.2. Data and preprocessing

We used terrain, climate, and vegetation properties over the study domain to model spatial variations of λ. An overview of the dataset used is presented in Table 1. For the terrain properties, we used predictors covering (i) groundwater as a secondary water resource, (ii) topographic complexity as a terrain property that modulates the amount of plant available water by lateral redistribution and convergence of soil moisture, and (iii) soil hydraulic properties as the fundamental modulator of available water and its accessibility by plants.

**Table 1:** Summary of the datasets used in the study.

**Variable**	**Data source**	**Spat. Res**.
Seasonal decay rate of vegetation cover (λ)	Küçük et al., 2022	5 km
Water Table Depth (WTD)	Fan et al., 2013	1 km
Height Above Nearest Drainage (HAND)	Yamazaki et al., 2019	90 m
Wetlands	Tootchi et al., 2019	500 m
– – – – – – – – – – – – – – – – – – – – – – – – – – – – – – – – – – – – – – – – – – – – – – – – – – – – – – – – – – – – – – –		
Topographic Wetness Index (TWI)		
Vectoral Ruggedness Measure (VRM)		
Magnitude and scale of 3D roughness	Amatulli et al., 2020	250 m
– – – – – – – – – – – – – – – – – – – – – – – – – – – – – – – – – – – – – – – – – – – – – – – – – – – – – – – – – – – – – – –		
Plant Available Water^a^ (PAW)		
Soil hydraulic conductivity at Field Capacity^a^ (*k*_*FC*_)	Estimated	
Max potential upwards capillary flux^a^^,^^b^ (*I*_*cap*_)		250 m
Precipitation^c^		
Temperature^c^	Fick and Hijmans, 2017	
– – – – – – – – – – – – – – – – – – – – – – – – – – – – – – – – – – – – – – – – – – – – – – – – -		
Shortwave Radiation^c^	Abatzoglou et al., 2018	5 km
Canopy height	Simard et al., 2011	1 km
– – – – – – – – – – – – – – – – – – – – – – – – – – – – – – – – – – – – – – – – – – – – – – – – – – – – – – – – – – – – – – –		
Tree and non-tree vegetation cover	Dimiceli et al., 2015	
Burned area	Giglio et al., 2015	
Plant Functional Type	Friedl and Sulla-Menashe, 2019	250 m

a Estimated using Hengl et al. (2017), based on Saxton and Rawls (2006).

b Based on Richards (1931).

c Annual and seasonal scales.

Solid lines are used to separate the variables as the target to model as well as the predictor groups of terrain, climate, and vegetation.

We defined the first set of terrain predictors considering Water Table Depth (WTD). In addition to the WTD data from Fan et al. (2013), we used Height Above Nearest Drainage (HAND) data from Yamazaki et al. (2019) that was generated using the MERIT digital elevation model at a spatial resolution of 90 m. HAND is useful to diagnose WTD variations as it is a good proxy to show the drainage positions (Fan et al., 2019), which strongly affect the groundwater table depth. We aggregated WTD and HAND by taking the arithmetic mean to have these data products at the same spatial resolution as λ. Even though seasonal variations of WTD may be significant, time series of high spatial resolution WTD is not available over large domains owing to the scarcity of observations and difficulties of modeling. Therefore, the WTD data used in this study is static and represents a climatological mean. As a proxy for regions with seasonally shallow groundwater, e.g., due to seasonal flooding, we used the wetlands data from Tootchi et al. (2019). The wetlands data was aggregated to target spatial resolution by computing the percentage of wetland area over target grid cells.

The second set of terrain predictors is related to topographic complexity. We used Topographic Wetness Index (TWI) as a proxy for the likelihood of lateral convergence of soil moisture. In order to account for slope and aspect at hillslope scales, we used Vectoral Ruggedness Measure (VRM) which is a compound metric quantifying slope and aspect together. The VRM values range from 0 to 1 and increase with topographic ruggedness. Finally, we used the magnitude and scale of terrain roughness, which is derived using VRM. The magnitude of roughness is an important parameter to represent the variation in topography even after spatial aggregation. All data of topographic complexity were derived by Amatulli et al. (2020) using the MERIT digital elevation model at 90 and 250 m resolutions. We used the data with 250 m resolution after aggregating to the target resolution (5 km) using the arithmetic mean.

In order to prepare the last set of terrain predictors, we used sand, clay, and organic matter contents of soil, and volumetric coarse fragments data from the SoilGrids dataset (Hengl et al., 2017) for top and deep soil. First, the SoilGrids dataset was aggregated (averaged) to the target resolution, which is the native resolution of λ (5 km). After grouping the layers up to 1 m as top soil and the rest as deep soil, we used the mean over layers as representative for top and deep soil. We then calculated soil hydraulic properties using the pedo-transfer functions provided in Saxton and Rawls (2006). Additionally, we estimated the maximum potential upward capillary flux (*I*_*cap*_) in millimeters per day (mm/day) assuming a fixed distance of 1 meter above the groundwater table using Richards' equation (Richards, 1931) for a 1-dimensional vertical soil column. Finally, we used Plant Available Water (PAW) as the difference between soil water content at field capacity and wilting point, soil hydraulic conductivity at field capacity (*k*_*FC*_), and *I*_*cap*_ for the top and deep soil layers as predictors to model λ.

In order to include climate characteristics as the predictors of the model, we used precipitation, temperature, and shortwave radiation data across annual and seasonal time scales. First, for all of the climate variables, we used a multi-annual mean as the predictor. In addition, several variables related to seasonal variation of precipitation and temperature were obtained from the WorldClim dataset (Fick and Hijmans, 2017). Finally, the seasonality of shortwave radiation was derived from the monthly TerraClimate dataset (Abatzoglou et al., 2018) by following the same approach used in Fick and Hijmans (2017). Spatial aggregation was not necessary for climatological predictors as the climate variables are at the same resolution as the target variable, λ, i.e., 5 km.

The last set of predictors covers vegetation characteristics. First, we used canopy height from Simard et al. (2011) after aggregating the data to 5 km resolution. Additionally, we used four MODIS based products related to vegetation: vegetation cover for tree and non-tree fractions (Dimiceli et al., 2015), burned area (Giglio et al., 2015), and Plant Functional Type (PFT) (Friedl and Sulla-Menashe, 2019). While the first three variables are aggregated by doing arithmetic mean over the target grid cell, the PFT, which is a categorical variable of types, is aggregated by using mode (most common type) over the target grid cell. In addition, we computed Shannon's diversity index (Shannon, 1948) of the PFTs within the target grid cells to represent the local scale variability of PFTs.

After preparing the data for use in modeling, we filtered out all the regions with annual precipitation larger than 1,500 mm/year. This filtering is necessary to only consider the drylands, as the spatiotemporal variations of λ in humid regions are associated with other confounding factors in addition to water limitation. To further reduce the uncertainties, we excluded regions with low confidence in λ values by filtering out regions with a relative standard error greater than 1, and with less than 3 successful convergences out of 16 estimations per grid cell (refer to Küçük et al., 2022 for details). Overall, around 7,30,000 grid cells with ca. Five kilometer spatial resolution were selected for the analysis presented in this study.

### 2.3. Methods

We used XGBoost (Chen and Guestrin, 2016), recent implementation of gradient boosted regression trees, to model spatial variations of λ using terrain, climate, and vegetation properties as predictors. Gradient boosting is an ML method that uses an ensemble of tree-based models generated by subsets of the training data. Tree based regression is a powerful method with high flexibility, designed to minimize output error with a strong gradient search without considering the underlying processes between predictors and target. In order to avoid unlikely attributions to predictors about the variation of λ, and ensure that the model consistently reflects the central assumption that secondary water buffers water-limited vegetation decay, we constrained the model to have a monotonic relationship between λ and terrain parameters. We essentially assume that any terrain property that promotes secondary water *via* additional moisture inputs should correlate positively with λ. Except with WTD and HAND, we constrained the model to have positive monotonicity between λ and terrain parameters, i.e., the larger the plant available water, the slower the vegetation decay. With WTD and HAND, negative constraints were set, i.e., the deeper the groundwater, the weaker its support to surface soil moisture. After setting the constraints, we used 10% of the grid cells, which are randomly selected, to build the model, and used the rest of the grid cells for validation.

Although tree based models are relatively easy to interpret, it is not trivial to estimate the importance of predictors of a multi-dimensional and nonlinear ML model in an unbiased way. Lundberg and Lee (2017) suggested using SHapley Additive exPlanation (SHAP) values to address the problem, which is rooted in cooperative game theory (Shapley, 1953) and treats each predictor as a player of a game. Being an additive explanation method, the summation of SHAP values of all predictors, e.g., for a grid cell, is equal to the deviation of the predicted value of that instance from the mean value of the predictions. Moreover, it is possible to partition the SHAP values for direct and interaction effects. In other words, for a simple modeling scenario of *y*_*obs*_ ≈ *y*_*m*_ = *f*(*x*_1_, *x*_2_) where *y*_*obs*_ and *y*_*m*_ are the observed and modeled target variable, and *x*_1_ and *x*_2_ are the predictors, $y_{m}=\bar{y_{m}}+\phi_{x1-x1}+\phi_{x2-x2}+\phi_{x1-x2}$ where $\bar{y_{m}}$ is mean of *y*_*m*_, ϕ_*x*1−*x*1_ and ϕ_*x*1−*x*2_ are the SHAP values attributed to predictor *x*_1_ alone and the interaction effects between the two predictors, respectively. Lundberg et al. (2020) suggested exploiting the model structures of tree based models to approximate SHAP values to avoid computational complexity on large datasets. In order to limit methodological problems related to feature interdependence (refer to Section 3.4) and improve interpretability, we grouped SHAP values of the predictors as terrain, climate, and vegetation properties, to explain the model output as:


(1) $$\begin{array}{rcl} \lambda & \approx & \lambda_{m}=\bar{\lambda_{m}}+\phi_{terrain-direct}+\phi_{terrain-clim}\\ +\phi_{terrain-veg}+\phi_{clim-direct}+\phi_{clim-veg}+\phi_{veg-direct} \end{array}$$

In order to quantify the importance of land parameters, we normalized the ϕ values of different sets of predictors after taking absolute values as:


(2) $$\begin{array}{l} \Phi_{terrain-total}=\frac{\left|\phi_{terrain-direct}\right|+\;\left|\phi_{terrain-clim}\right|+\;\left|\phi_{terrain-veg}\right|}{\left|\phi_{terrain-direct}\right|+\;\left|\phi_{terrain-clim}\right|+\;\left|\phi_{terrain-veg}\right|+\;\left|\phi_{clim-direct}\right|+\;\left|\phi_{clim-veg}\right|+\left|\phi_{veg-direct}\right|} \end{array}$$

Finally, we analyzed the variations of Φ_*terrain*−*total*_ with changing WTD, VRM, and *I*_*cap*_ values and the sensitivity of these covariations against annual precipitation.

## 3. Results

### 3.1. Model output for seasonal decay rate of vegetation cover

**Figure 1:**
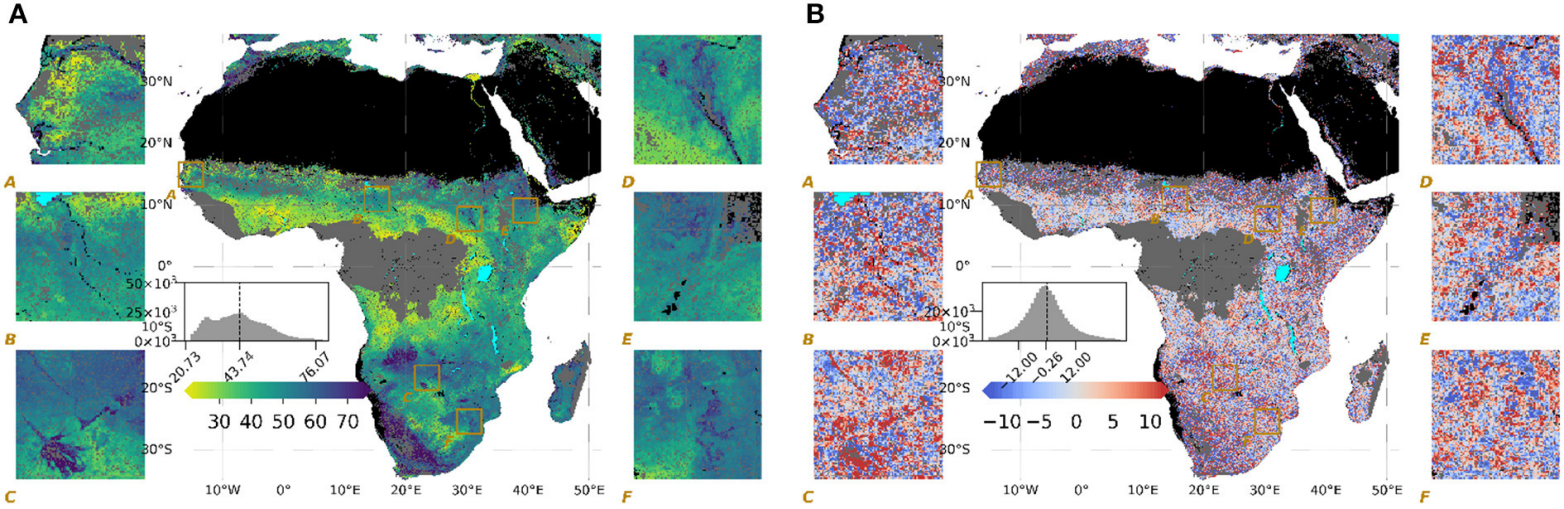
Maps of **(A)** model output (λ_*m*_), in days, where larger values of λ (blue) indicate slower decay **(B)** residual of the model (λ−λ_*m*_), in days, where positive values (red) indicate underestimation. Histograms of the mapped values for the entire domain are given in the main panels of all the maps with a dashed line indicating the mean values of the domain, as well as six insets to show local variability.

The ML model captured the continental gradient as well as local variations of λ (λ_*m*_, shown in Figure 1A) with 55% Nash–Sutcliffe modeling efficiency (Nash and Sutcliffe, 1970). We consider this a satisfactory achievement given the complexity of processes shaping vegetation decay patterns and the monotonic constraints in terrain predictors to reflect the central assumption of the model. The residuals of the model show anisotropic structures at local scales (Figure 1B), suggesting that the model did not capture all the local-scale variations, presumably due to incomplete and imperfect predictors (refer to Section 3.4 for further discussion).

In the following, we use the trained model to attribute and analyze the contribution of predictors associated with secondary water effects on λ_*m*_ based on SHAP values. By nature, this analysis exploits the patterns of λ_*m*_ variations that are explained by the ML model.

### 3.2. Importance of terrain properties on seasonal decay rate of vegetation cover

**Figure 2:**
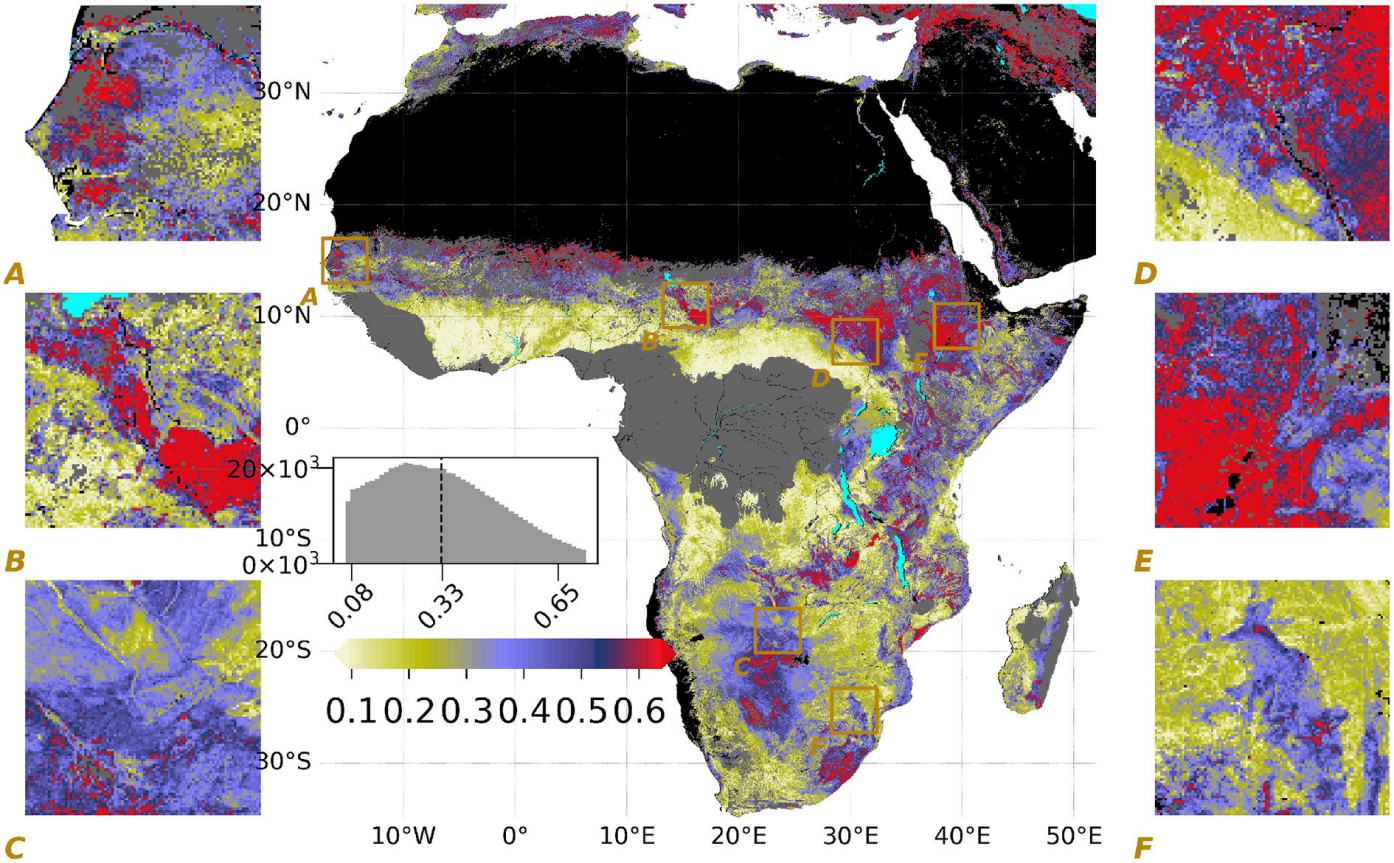
Spatial variations of the normalized importance of terrain on λ (Φ_*terrain*−*total*_) as the output of Equation 2 where larger (blue to red) values indicate higher importance of terrain parameters. Refer to Figure 1 for plotting details.

Based on SHAP values, the spatial variation of the normalized importance of terrain λ (Φ_*terrain*−*total*_, refer to Equation 2) is shown in Figure 2 together with six zoomed insets and a histogram of the values, in which the mean value over the domain is shown with a dashed line. Over the study domain, 33% of the variation in λ is attributed to terrain effects on average, 17% of which are direct effects, and 16% are from the interactions with climate and vegetation properties.

Moreover, we find hotspots where the importance of terrain properties λ is larger than 60% (Figure 2) with complex and structured spatial patterns. These patterns agree well with estimates of the importance of evaporation from secondary water sources using a hydrological model that assimilates different remote sensing data (Van Dijk et al., 2018).

Some regions with shallow groundwater are within these hotspots, such as Box-B showing the South of Lake Chad, between the Logone and Chari Rivers and the Sudd Swamp—Figure 2 (refer to Fan et al., 2013 for water table depth estimates), consistent with previous studies on the relevance of shallow water tables for vegetation activity in water-limited environments (Koirala et al., 2017; Roebroek et al., 2020).

Furthermore, we found strong terrain effects over mountainous regions such as the Ethiopian Highlands (Box-E) and, to a lesser extent, the Manica Highlands (Box-F) (refer to Clark et al., 2017 for further information about the Manica Highlands). This likely reflects the effects of topographical complexity on lateral water flows and moisture convergence in valleys and riparian zones (Fan et al., 2019).

**Figure 3:**
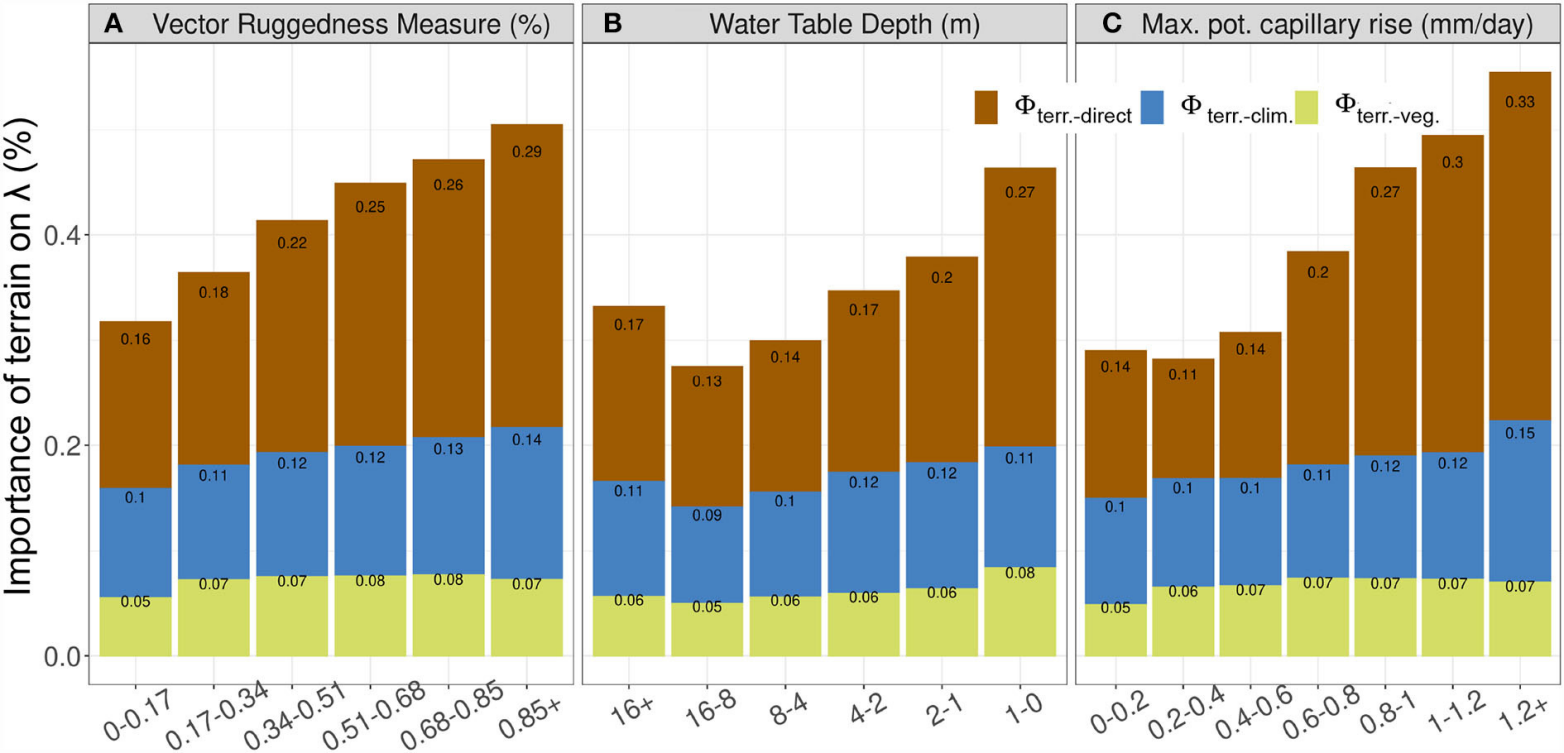
Normalized importance of terrain (same as Figure 2) with change in Vector Ruggedness Measure (VRM) **(A)**, Water Table Depth (WTD) **(B)**, and maximum potential upwards capillary flux 1 m above water table depth (*I*_*cap*_) **(C)**. Y-axis shows the total terrain effects (Φ_*terrain*−*total*_) even though bars are colored and annotated to show its components as direct effects (Φ_*terrain*−*direct*_) and interaction effects with climate (Φ_*terrain*−*clim*_) and vegetation (Φ_*terrain*−*veg*_), using Equation 2.

Now, we inspect how the importance of terrain properties varies with individual terrain variables related to topographic complexity, groundwater, and capillary rise, respectively. Pooled over the entire study domain, we found that the importance of terrain properties increases systematically with VRM, a metric summarizing topographic complexity (Figure 3A). About half of the importance of terrain properties is associated with VRM values above 0.85. Lateral water flows and moisture convergence in complex terrain enhance the relevance of secondary water effects on vegetation as previously reported at the watershed scale (Hoylman et al., 2018; Tai et al., 2020).

The effect of terrain properties on λ increases systematically with shallower water table depth (Figure 3B). Regions with water table depths <1 m are associated with almost half of the importance of terrain properties. This effect is gradually reduced with deeper groundwater levels up to 16 m. This relation, however, does not hold at WTD levels deeper than 16 m, presumably due to the disconnection between surface and groundwater, where other factors become more prominent. The local increase of importance at water tables deeper than 16 m is due to covariation with high topographic complexity in mountainous regions (refer above). Our findings suggest that shallow water tables are an important secondary water source for vegetation across Africa, consistent again with the previous findings (Madani et al., 2020).

We also observed a systematic increase in the importance of terrain properties with increasing propensity for capillary rise *I*_*cap*_ associated with soil texture properties (Figure 3C). Overall, more than half of the importance of terrain for λ is attributed when *I*_*cap*_ > 1 mm/day. This suggests that enhancement of soil moisture due to the presence of vertically upward capillary flux plays an important role in providing secondary water for vegetation activities across most parts of Africa. This is generally consistent with previous studies identifying soil texture as a key variable mediating the interactions between climate, soil, and vegetation (Fernandez-Illescas et al., 2001).

### 3.3. Effects of aridity on the importance of terrain parameters

**Figure 4:**
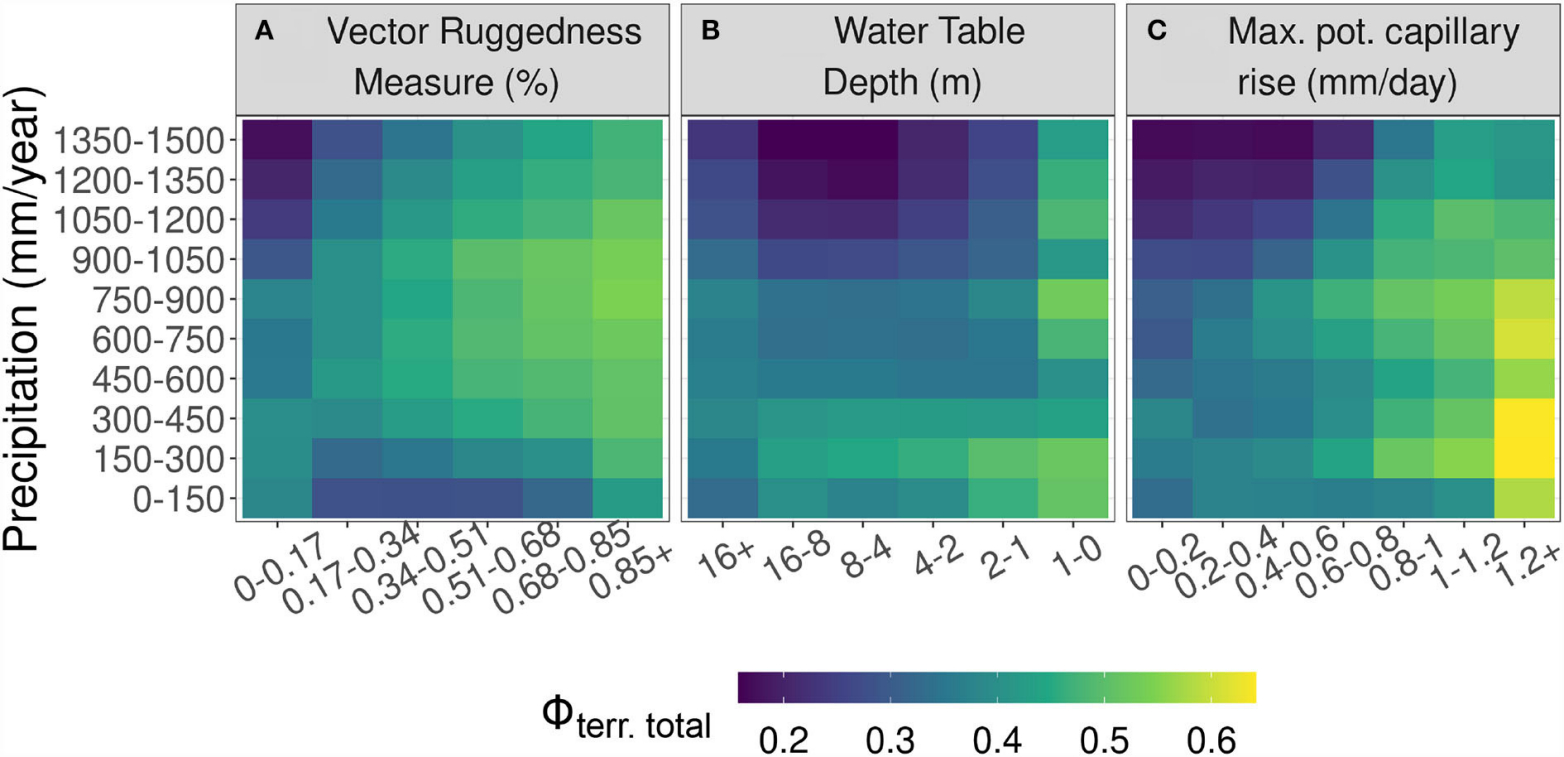
Effects of aridity on the importance of terrain parameters (refer to Equation 2) with change in Vector Ruggedness Measure (VRM) **(A)**, Water Table Depth (WTD) **(B)**, and maximum potential upward capillary flux 1 meter above water table depth (*I*_*cap*_) **(C)**.

Since secondary water effects are contingent on the supply of rainfall, we analyze how the importance of terrain variables on λ covaries with VRM, WTD, and *I*_*cap*_ over a precipitation gradient of 0–1,500 mm/year (Figure 4A). The positive relationship between Φ_*land*−*total*_ and VRM is generally consistent across different precipitation regimes. The gradient between low and high topographic complexity, though, is more pronounced in wet and dry regimes compared to the gradient at intermediate precipitation. The larger sensitivity of Φ_*land*−*total*_ to topographic complexity under wetter conditions likely reflects more secondary water effects due to lateral flows of excess rainfall. Under very dry conditions, topographic complexity needs to be relatively large to have a sizable effect on secondary water, primarily because most of the rainfall would be lost through evaporation (Newman et al., 2006), therefore, not producing a significant excess percolation needed for lateral sub-surface flows. Thus, at intermediate topographic complexity, the degree of secondary water effects peaks at intermediate aridity. The optimality of intermediate conditions in enhancing the role of groundwater moisture sources has also been reported previously (Koirala et al., 2014).

Shallow water tables (<1 m) are associated with high importance of Φ_*land*−*total*_ across all precipitation regimes suggesting strong secondary water effects by groundwater when easily accessible (Figure 4B). As water tables get a bit deeper with depths of a few meters, there is a tendency of increasing Φ_*land*−*total*_ with increasing aridity suggesting that secondary water supply by groundwater gains relevance as the primary water supply by rainfall decreases (Brooks et al., 2015).

The positive relationship between *I*_*cap*_ and the importance of terrain variables on λ is consistent across precipitation regimes, while the effects get stronger with increasing aridity (Figure 4C) except for the most dry, hyper-arid conditions. These patterns suggest an important role of soil mediated capillary rise as a secondary water effect for vegetation, in particular in regions of intermediate aridity.

### 3.4. Robustness and limitations

The machine learning based quantification and analysis of the effects of secondary water on the seasonal vegetation decay over Africa have uncertainties associated with the underlying assumptions and methods. The assumption that vegetation decay is primarily due to moisture limitation in most African ecosystems is supported by previous studies (refer to Küçük et al., 2022 and references therein). In order to limit the uncertainty, we confined the study domain to retain primarily water-limited systems by excluding the humid tropical regions (refer to Section 2.2). The key findings of our study on the importance and patterns of land characteristics associated with secondary water for vegetation decay are consistent with the assumption that African drylands are primarily water-limited.

The main methodological uncertainties are related to i) the quality and performance of the underlying trained machine learning model and ii) the correct attribution of modeled λ variations to terrain properties. Regarding the quality and performance of the model, we acknowledge that the XGBoost model explained only 55% of λ variations based on training on only 10% of randomly selected pixels to avoid overfitting due to spatial auto-correlation (Roberts et al., 2017). While this performance seems relatively low at the first glance, explaining the majority of the variance can be considered an important achievement given the complexity of processes shaping vegetation decay patterns, accompanied by very little knowledge about underlying mechanisms and processes. However, it also suggests that we are lacking predictors and/or that there are inherent issues in the quality of data products used as predictors. The imperfect representation of terrain factors governing secondary water in the predictor set is likely constrained further by the spatial resolution of 3–5 km where important sub-grid variations of factors and responses in λ may not be resolved adequately. The model residuals (Figure 1B) show clear spatial structures at meso-scales but not at large scales. Thus, we likely underestimate λ variations due to landscape-scale factors which suggest that the spatial patterns and attribution of the importance of terrain properties are probably conservative and that these may be even stronger at higher spatial resolution.

The machine learning based attribution of vegetation decay patterns to terrain variables is based on the interpretation of the trained model and, therefore, on those patterns that are captured and explained by the model. We acknowledge that ML methods exploit statistical associations without any guarantee of unraveling causal relationships. In our experimental design, we aimed at enhanced interpretability of the results by constraining the predictor set to interpretable factors related to our objectives, and by constraining the monotonicity of terrain predictors to λ according to prior knowledge. Note that the monotonic constraints prescribe only the sign of the response which acts as a causal regularization in the model training process, but the shape remains flexible. Some confidence in the qualitative findings of the study originates from the fact that the revealed importance of terrain properties varies systematically with topographic complexity, water table depth, and maximum capillary rise, and these are largely consistent with understandings from theory and previous studies (Figure 3). We would like to note that the result and findings presented here are not trivial and are not enforced by the monotonic predictor constraints since the terrain importance was estimated as mean absolute deviations (Equation 2).

We used SHAP values as the state-of-the-art technique for machine learning based attribution to predictors, while attribution in the presence of large covariations among predictors remains a challenge Kumar et al. (2020). We aimed at minimizing this issue by analyzing the importance of predictor groups, rather than individual predictors based on the consistent aggregation of SHAP values (refer to Equations 1 and 2). This makes our attribution robust against covariation of predictors within a group, e.g., within the terrain group of predictors. We assume that most of the covariation among predictors is within the group, but there remains covariation across groups that can potentially lead to some confounding effects.

Finally, the main challenge of our study design is that it is nearly impossible to validate the findings quantitatively using independent observations. On a qualitative comparison, we find patterns that are consistent with understandings from theory and previous studies. Nevertheless, we encourage future studies to consider the patterns of secondary moisture effects on vegetation revealed here as a new hypothesis inferred from machine learning, that should be scrutinized and tested by complementary tools and methods.

## 4. Discussion

In this study, we explored the effects of local scale secondary water associated with climate, terrain, and vegetation characteristics on seasonal vegetation decay rate (λ) of fractional vegetation cover over African drylands at 5 km spatial resolution based on machine learning methods. We find that the importance of terrain properties for λ can be larger than 60% in certain hotspot regions. Over the full study domain of Africa, the importance of terrain properties is on average 33%, which is about equally split among direct effects of terrain properties and interaction effects of terrain properties with climate and vegetation properties. The importance of terrain effects on λ increases with aridity, suggesting an increasing role of secondary water effects on vegetation. We further find that the importance of terrain properties on vegetation decay increases with increasing maximum potential capillary rise determined by soil texture properties (*I*_*cap*_), as the ground water tables become shallow. The patterns become stronger with increasing aridity, presumably highlighting a large role of secondary water under such conditions. The importance of terrain properties on vegetation decay also increases with topographic complexity, with the strongest patterns in regions of intermediate aridity where the complex topographic condition with optimal precipitation input leads to regions with water table depth that can still be accessed by vegetation. Our observation-based study suggests that local scale processes affecting water availability in drylands have widespread and significant relevance over the continental domain in Africa, and these processes cannot be neglected. The presented patterns of topographic variability, water table depth, and soil propensity to capillary rise on dry season vegetation decay can help guide the development of global land models to account for the effects of secondary water. Incorporating these mediating terrain effects on drought responses in Earth System models may have large implications for simulated ecosystem processes such as the carbon cycle, water turnover timescales, and, consequently, land-atmosphere feedbacks.

## Data availability statement

The datasets used for this study can be found in the cited literature (summarized in Table 1). Raster files of the normalized importance of terrain effects as well as the raw SHAP values of direct and interaction effects of terrain, climate, and vegetation are available in https://doi.org/10.6084/m9.figshare.16780405.v2.

## Author contributions

ÇK and MJ contributed to the conception and design of the study. ÇK performed the experiments and the analyses with support from MJ for computation of SHAP values, and SK for estimation of soil hydraulic properties. ÇK wrote the first draft of the manuscript. All the authors provided intellectual contributions during the analyses of the results, contributed to manuscript revision, read, and approved the submitted version.

## Funding

ÇK acknowledges funding from the International Max Planck Research School for Global Biogeochemical Cycles. SK acknowledges the support of the Erdsystemforschung: Afrikanische Grundwasserressourcen im Zuge des globalen Wandels (Earth System Research: Groundwater Resources in Africa under Global Change) project of the Max Planck Society. DM acknowledges funding from the European Research Council (ERC) under grant agreement 715254 (DRY-2-DRY) and the European Union Horizon 2020 Programme project 869550 (DOWN2EARTH). MR acknowledges funding by the European Research Council (ERC) Synergy Grant Understanding and modeling the Earth System with Machine Learning (USMILE) under the Horizon 2020 research and innovation program (Grant Agreement No. 855187).

## Conflict of interest

The authors declare that the research was conducted in the absence of any commercial or financial relationships that could be construed as a potential conflict of interest.

## Publisher's note

All claims expressed in this article are solely those of the authors and do not necessarily represent those of their affiliated organizations, or those of the publisher, the editors and the reviewers. Any product that may be evaluated in this article, or claim that may be made by its manufacturer, is not guaranteed or endorsed by the publisher.
