# Supplementary material for: Observation-based assessment of secondary water effects on seasonal vegetation decay across Africa
Source: Front Big Data. 2022 Sep 9;5:967477. doi: 10.3389/fdata.2022.967477 (PMC9500241; doi:10.3389/fdata.2022.967477)
Supplement: Supplementary file 1 [file Data_Sheet_1.PDF]

Supplementary Information for “Observation-based assessment of secondary water effects on seasonal vegetation decay across Africa”

1 SPATIAL DISTRIBUTION OF THE TARGET VARIABLE

Spatial distribution of the target variable is shown in figure S1. Yellow color represents faster rate of seasonal vegetation cover decay while blue represents slower decay. Note that study domain is filtered based on the quality of λ estimations and annual precipitation of 1500 mm/year. Further details of the metric and its derivation is available in Küçük et al. (2022).

2 TERRAIN ATTRIBUTED VARIATIONS IN λ

In this section, we present the terrain attributed variations of λ using the raw SHAP values of the modeled λ. Spatial variation of the raw SHAP values are given in figure S2 as total attribution, together with it's component as direct terrain effects and terrain interaction effects with climate and vegetation in the panels. Directly terrain attributed variations ($\phi_{terrain-direct}$) are the dominant component of the total terrain attributed variations of λ ($\phi_{terrain-total}$). Large positive values in $\phi_{terrain-direct}$ in regions with shallow groundwater like the Sudd Swamp (Box-D) where groundwater is shallow (Tootchi et al., 2019) and with complex topography like the Ethiopian Highlands (Box-E) show that the e-folding time of FVC is slowed down up to 6 days directly owing to the terrain parameters modulating secondary water resources – see figure S2A. Conversely, we observed strong negative effects in very arid regions like Senegal (Box-A in figure S2B), Somalia, and the Kalahari Desert, where groundwater is disconnected from surface (Fan et al., 2013).

Interestingly, interaction effects between terrain and climate make strong positive variation on λ in Okavango Delta (Box-C in figure S2C) that inverts the negative effects of terrain parameters in the region. This conceptually agrees with the fact that the Okavango Delta, being a seasonally flooded delta, is strongly affected by climate seasonality (Cronberg et al., 1995). Lastly, interaction effects between terrain and vegetation are not so prominent through the study domain (figure S2D).

3 CLIMATE AND VEGETATION ATTRIBUTED VARIATIONS IN λ

Spatial variations of raw SHAP values for climate and vegetation are given in figure S3 to illustrate their effects on λ as direct effects ($\phi_{clim-direct}$ and $\phi_{veg-direct}$) and interaction effects ($\phi_{clim-veg}$).

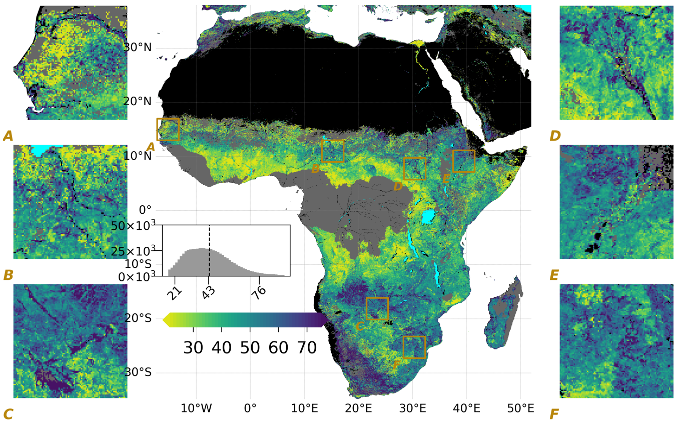

Figure S1. Observed λ as the target variable of the gradient boosting model.

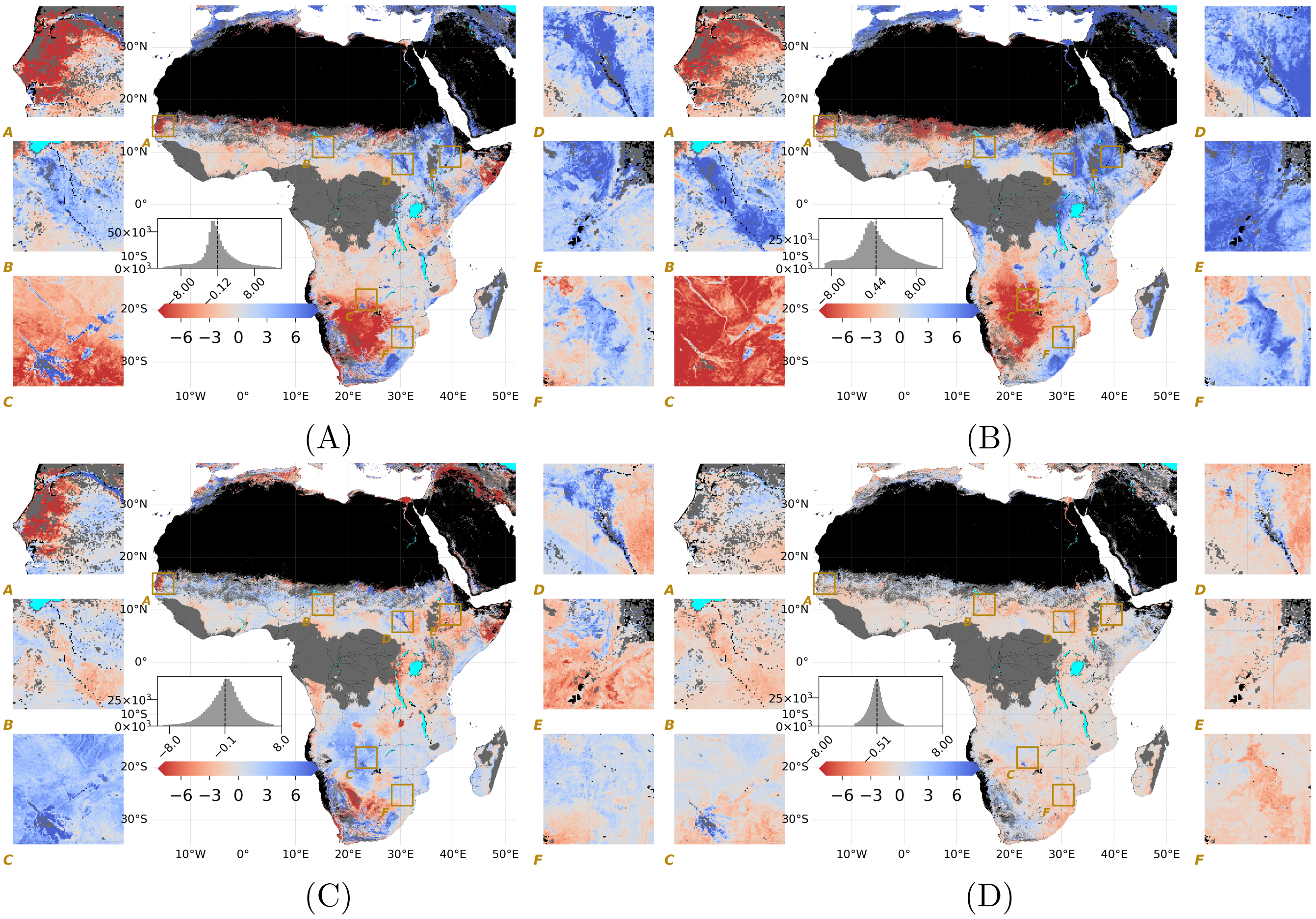

Figure S2. Terrain attributed variations of λ as **(A)** total effects, $\phi_{terrain-total} = \phi_{terrain-direct} + \phi_{terrain-clim} + \phi_{terrain-veg}$, **(B)** direct effects of terrain, $\phi_{terrain-direct}$, **(C)** interaction effects between terrain and climate, $\phi_{terrain-clim}$, **(D)** interaction effects between terrain and vegetation, $\phi_{terrain-veg}$.

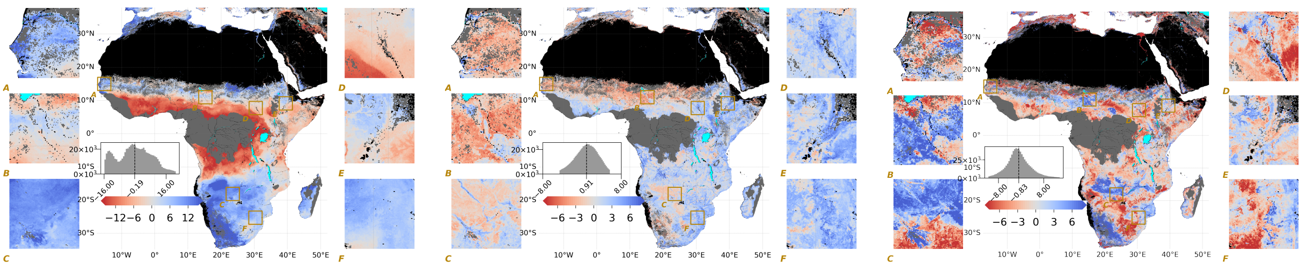

(A) (B) (C)

Figure S3. Maps of feature attribution for **(A)** direct effects of climate ($\phi_{clim-direct}$), **(B)** direct effect of vegetation ($\phi_{veg-direct}$), **(C)** interaction effects between climate and vegetation ($\phi_{clim-veg}$). Note the larger range of colorbar in $\phi_{clim-direct}$ than other maps of raw SHAP values.

REFERENCES

Cronberg, G., Gieske, A., Martins, E., Prince Nengu, J., and Stenström, I.-M. (1995). Hydrobiological studies of the Okavango Delta and Kwando/Linyati/Chobe River, Botswana I surface water quality analysis. *Botswana Notes and Records* 27

Fan, Y., Li, H., and Miguez-Macho, G. (2013). Global patterns of groundwater table depth. *Science* 339, 940–943. doi:10.1126/science.1229881

Küçük, Ç., Koirala, S., Carvalhais, N., Miralles, D. G., Reichstein, M., and Jung, M. (2022). Characterizing the response of vegetation cover to water limitation in africa using geostationary satellites. *Journal of Advances in Modeling Earth Systems* 14. doi:10.1029/2021MS002730

Tootchi, A., Jost, A., and Ducharne, A. (2019). Multi-source global wetland maps combining surface water imagery and groundwater constraints. *Earth System Science Data* 892657, 189–220. doi:10.5194/essd-11-189-2019
